# Urogenital function in robotic vs laparoscopic rectal cancer surgery: a comparative study

**DOI:** 10.1007/s00384-016-2682-7

**Published:** 2016-10-21

**Authors:** Sofoklis Panteleimonitis, Jamil Ahmed, Meghana Ramachandra, Muhammad Farooq, Mick Harper, Amjad Parvaiz

**Affiliations:** 1 Department of Minimally Invasive Colorectal Unit, Queen Alexandra Hospital NHS Trust, Portsmouth, UK; 2 School of Health Sciences and Social Work, University of Portsmouth, Portsmouth, UK; 3 Colorectal Cancer Unit, Champalimaud Clinical Foundation, Lisbon, Portugal

**Keywords:** Robotic surgery, Rectal cancer, Urogenital function, Urological function, Sexual function

## Abstract

**Purpose:**

Urological and sexual dysfunction are recognised risks of rectal cancer surgery; however, there is limited evidence regarding urogenital function comparing robotic to laparoscopic techniques. The aim of this study was to assess the urogenital functional outcomes of patients undergoing laparoscopic and robotic rectal cancer surgery.

**Methods:**

Urological and sexual functions were assessed using gender-specific validated standardised questionnaires. Questionnaires were sent a minimum of 6 months after surgery, and patients were asked to report their urogenital function pre- and post-operatively, allowing changes in urogenital function to be identified. Questionnaires were sent to 158 patients (89 laparoscopy, 69 robotic) of whom 126 (80 %) responded. Seventy-eight (49 male, 29 female) of the responders underwent laparoscopic and 48 (35 male, 13 female) robotic surgery.

**Results:**

Male patients in the robotic group deteriorated less across all components of sexual function and in five components of urological function. Composite male urological and sexual function score changes from baseline were better in the robotic cohort (*p* < 0.001). In females, there was no difference between the two groups in any of the components of urological or sexual function. However, composite female urological function score change from baseline was better in the robotic group (*p* = 0.003).

**Conclusion:**

Robotic rectal cancer surgery might offer better post-operative urological and sexual outcomes compared to laparoscopic surgery in male patients and better urological outcomes in females. Larger scale, prospective randomised control studies including urodynamic assessment of urogenital function are required to validate these results.

## Introduction

Rectal cancer surgery is associated with a high risk of urological and sexual dysfunction which significantly affects the quality of life of its survivors [1–6]. Although urogenital dysfunction is thought to be multifactorial in nature, intra-operative damage to the autonomic pelvic nerves is considered to be the primary cause [5–7].

It is argued that better visualisation of the pelvic autonomic nerves such as obtained during minimally invasive surgery could enable the preservation of these structures and therefore reduce the incidence of urogenital dysfunction [8]. However, despite laparoscopic total mesorectal excision (TME) becoming the standard approach in much of the modern world, it is still debated whether it has helped improve urogenital dysfunction. In fact, the evidence comparing the urogenital outcomes of open and laparoscopic TME is conflicting, with some studies advocating favourable urogenital outcomes for laparoscopic TME [9] whilst others for open TME [10]. A recently published systematic review concluded that neither approach is superior in terms of urogenital preservation [11]. A probable explanation for this is that laparoscopic rectal surgery is technically difficult [12]. Existing laparoscopic instruments have a restricted range of movement compared with those of the surgeons’ hand and are difficult to use in confined spaces such as the pelvis [13, 14].

Robotic surgical systems were introduced to overcome the technical limitations of laparoscopic surgery [15]. With superior three-dimensional views, tremor filtering and angulated instruments, robotic surgery enables precise dissection in narrow surgical spaces such as the pelvis therefore enabling better preservation of fine structures such as the autonomic nerves [13, 16]. Currently, there are only a few studies investigating the urological and sexual outcomes of robotic surgery against those of laparoscopic surgery and these tend to be predominant about male patients.

The aim of this retrospective clinical study is to compare the urological and sexual functional outcomes of robotic and laparoscopic rectal cancer surgery using a validated functional questionnaire in both men and women in a high-volume minimally invasive colorectal unit. Only one similar study has been identified and deemed up to date but its sample size is significantly smaller [17]; therefore, our study aims to build upon that evidence base.

## Methods

All patients who underwent potentially curative elective laparoscopic or robotic rectal cancer resections from December 2006 to September 2009 for the laparoscopic group and from May 2013 and September 2014 for the robotic group were identified from a prospectively maintained database. Rectal cancer was defined as cancer present within 15 cm from the anal verge. Surgery was performed in both groups by two surgeons with vast laparoscopic and robotic experience working in a high-volume minimally invasive colorectal unit. Urological and sexual function was assessed using gender-specific validated questionnaires sent a minimum of 6 months after surgery to allow for wound healing and objective data collection. Urogenital data for the laparoscopic group was collected during a previous study comparing the urogenital outcomes of laparoscopic and open rectal surgery [9]. Urogenital data for the robotic group was collected subsequently, when the two performing surgeons adopted its practice.

Formal oncological and physical assessment was undertaken by all patients prior to surgery following the protocol. Pre-operative staging was performed by colonoscopy or CT colonography, computed tomography (CT) of the chest and abdomen and magnetic resonance imaging (MRI) of the pelvis. Patients with low rectal cancers (5 cm from anal verge) underwent additional staging with endo-anal ultrasound (EUS).

All patient findings were discussed in the multidisciplinary team meeting prior to initiating any type of treatment. In general, pre-operative long-course chemoradiotherapy was reserved for T4 rectal cancers or those where the circumferential resection margin (CRM) appeared threatened on MRI, whilst short-course radiotherapy was advised for rectal cancers that approached but did not threaten the CRM. Radiotherapy was not used where rectal cancers were considered resectable by TME with a good likelihood of clear margins.

Appropriate approval for the study was obtained by the Research and Development department of Portsmouth NHS Trust. Informed consent was also obtained from the patients participating in this study.

### Surgical technique

The laparoscopic group had a standardised technique which has been previously published [9, 18]. A modular approach of medial to lateral colonic mobilisation with isolation and ligation of the main vessels using clips was applied, and TME was performed using monopolar diathermy as previously described [18].

Robotic rectal resections were performed using a single docking fully robotic approach [19]. The principle of standardised technique developed for laparoscopic surgery was also used for robotic surgery. Procedures commenced with medial to lateral dissection followed by vascular control by ligating the main vessels. Hem-o-loks® were used to secure the vessels before division and a three-step approach was used for splenic mobilisation [20]. TME was performed in the same stepwise manner as in the laparoscopic group, starting with posterior mobilisation followed by right lateral, anterior and left lateral mobilisation in a stepwise manner. Similarly, robotic dissection was performed using monopolar diathermy. Post-operatively, all patients were managed using the enhanced recovery program described by Kehlet and Wilmore [21]. Patients were discharged home according to set criteria for discharge.

### Patient selection

No specific selection criteria were used to allocate patients to laparoscopic or robotic surgery. In this study, two surgeons performed all laparoscopic and robotic resections. Between 2006 and 2009, laparoscopy was the preferred approach for rectal surgery, whereas following the adoption of robotic surgery in May 2013, robotic surgery became the surgical approach of choice for rectal cancers.

### Urogenital function assessment

Urogenital function was assessed as described in our previous study [9]. Anonymous and confidential questionnaires were sent to all surviving patients in February 2010 for the laparoscopic group and in May 2015 for the robotic group, a minimum of 6 months following surgery through the post. Patients were asked to rate their urological and sexual function pre-operatively and post-operatively. To maximise patient response rate, patients that did not reply to the questionnaires within 4 weeks were sent a reminder letter.

For male urological function, we used a modification of the International Prostatic Symptoms Score (IPSS) [22], for male sexual function the International Index of Erectile Function (IIEF-5) [23], for female urological function the King’s Health questionnaire [24] and for female sexual function the Female Sexual Function Index (FSFI) [25].

The following components were assessed in each questionnaire:

1. Male urological function (MUF): frequency, nocturia, urgency, straining, poor flow and incomplete bladder emptying.
2. Male sexual function (MSF): libido, erection, stiffness for penetration and orgasm/ ejaculation.
3. Female urological function (FUF): frequency, nocturia, urgency and stress incontinence.
4. Female sexual function (FSF): arousal/ libido, lubrication, orgasm and dyspareunia.

Overall, there were six components for MUF and four for MSF, FUF and FSF. Each component was analysed independently and a composite score for each questionnaire was created by adding the scores of each component. Scoring was standardised and quantified for all questions. The following scale was applied: 0 for not at all, 1 for less than half the time, 2 for about half the time, 3 for more than half the time and 4 for almost always. A score of 0 reflected normal function whilst 4 poor function. Overall, a high score reflected a high degree of dysfunction whereas a low score reflected normal function.

### Statistical analysis

IBM SPSS version 22 (SPSS Inc., Chicago, IL, USA) and Microsoft Excel 2010™ were used for the statistical analysis. Data was expressed as mean ± standard error of the mean and median with interquartile range for parametric and non-parametric data, respectively. Baseline demographic and clinical characteristics were compared using the *χ* ^2^ test or Fisher’s exact test for categorical variables and the *t* test or Mann-Whitney *U* test for continuous variables. Urogenital functional scores were compared using a *t* test. *p* values of <0.05 were considered statistically significant. Sexually inactive patients were excluded from the sexual outcome analysis to avoid skewing of the data.

## Results

### Patient characteristics

Questionnaires were sent to 158 patients (89 laparoscopic group, 69 robotic) of whom 126 (80 %) responded. Seventy-eight (49 male, 29 female) of the responders underwent laparoscopic and 48 (35 male, 13 female) robotic surgery. Of those, 45 patients (36 male, 9 female) were sexually active in the laparoscopic group and 17 (13 male, 4 female) in the robotic group.

The demographic, clinical and pathological characteristics of the patients included in this study are summarised in Table 1. The baseline characteristics of the two groups were broadly comparable. Nevertheless, patients in the robotic group had lower rectal cancers (*p* = 0.032) and were more likely to receive long-course pre-operative radiotherapy (*p* = 0.012) and neoadjuvant chemotherapy (*p* = 0.030).

**Table 1:** Baseline demographic and clinico-pathological features

	Laparoscopic	Robotic	*p* value
Gender			
•Male	49	35	0.243
•Female	29	13	
Age			
Median (IQR)	70 (63–75.25)	69 (64–74.75)	0.966
BMI median (IQR)			
•Male	26 (23–29.5)	27 (25–28.25)	0.275
•Female	26 (24–32.5)	27 (24.25–29.5)	1.000
ASA grade			
•1	12 (15 %)	4 (9 %)	0.407
•2	51 (65 %)	39 (85 %)	*0.019*
•3	15 (19 %)	3 (7 %)	0.066
Type of surgery			
❖Total AR	63 (81 %)	40 (84 %)	0.716
•Male AR	38 (76 %)	30 (86 %)	0.409
•Female AR	25 (86 %)	10 (77 %)	0.657
❖Covering ileostomy	56 (89 %)	34 (85 %)	0.562
•Male	36 (95 %)	26 (87 %)	0.394
•Female	20 (80 %)	8 (80 %)	1.000
❖Total APER	14 (18 %)	7 (15 %)	0.623
•Male	10 (20 %)	4 (11 %)	0.377
•Female	4 (14 %)	3 (23 %)	0.657
❖ Total Hartman’s	1 (1 %)	1 (2 %)	1.000
•Male	1 (2 %)	1 (3 %)	1.000
•Female	0	0	1.000
Anal verge mean (SE)	9.36 ± 0.38	7.91 ± 0.54	*0.032*
T stage			
•Tx	4 (5 %)	2 (4 %)	1.000
•T1	6 (8 %)	6 (13 %)	0.372
•T2	26 (33 %)	21 (44 %)	0.240
•T3	36 (46 %)	16 (33 %)	0.156
•T4	6 (8 %)	3 (6 %)	1.000
Radiotherapy			
•Pre-op short	9 (12 %)	0	*0.014*
•Pre-op long	5 (6 %)	11 (23 %)	*0.012*
•Pre-op total	14 (18 %)	11 (23 %)	0.497
•Post-operative	0	1 (2 %)	0.328
Chemotherapy			
•Neoadjuvant	7 (9 %)	11 (23 %)	*0.030*
•Adjuvant	18 (23 %)	16 (33 %)	0.208
Post-op complications			
•Anastomotic leak	3 (4 %)	4 (5 %)	1 (2 %)
•Return to theatre	0	1.000	0.301

### Male urological function

**Fig. 1:**
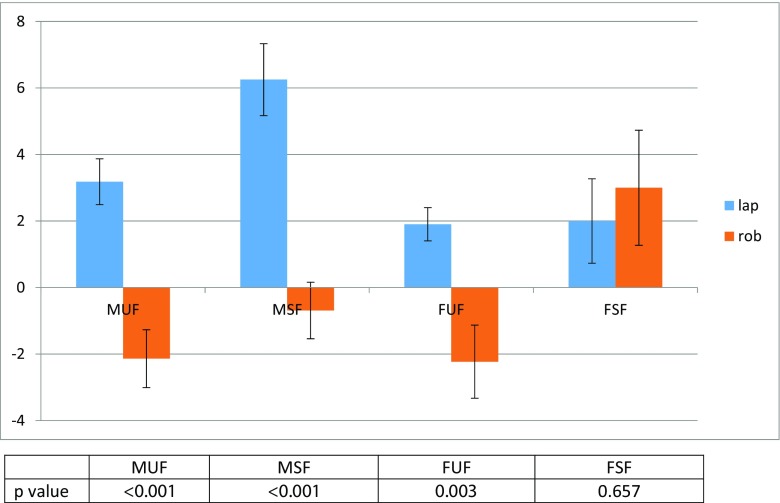
Change in mean composite scores from baseline

There were 49 patients in the laparoscopic group and 35 in the robotic group. In Tables 2 and 3 and Fig. 1, we present the mean pre-operative MUF scores and their change from baseline. Pre-operative urological function was worse across three components (frequency, nocturia, urgency) in the robotic group, and the pre-operative composite mean MUF score was worse in the robotic group.

**Table 2:** Baseline and change from baseline MUF mean scores (mean ± standard error of the mean)

	Laparoscopic	Robotic	*p* value
Baseline MUF			
•Frequency	1.63	2.51	*0.013*
•Nocturia	2.06	2.91	*0.013*
•Urgency	0.59	1.63	*0.003*
•Initiation/straining	0.16	0.26	0.576
•Poor flow	0.69	1.26	0.090
•Incomplete bladder emptying	0.92	1.20	0.406
Change from baseline			
•Frequency	0.57 ± 0.16	−0.31 ± 0.22	*0.002*
•Nocturia	0.63 ± 0.17	−0.20 ± 0.19	*0.002*
•Urgency	0.69 ± 0.21	−0.66 ± 0.29	*<0.001*
•Initiation/straining	0.39 ± 0.12	0.09 ± 0.13	0.094
•Poor flow	0.73 ± 0.18	−0.14 ± 0.21	*0.002*
•Incomplete bladder emptying	0.16 ± 0.20	−0.63 ± 0.26	*0.017*

**Table 3:** Mean composite MUF scores

	Pre-op	Post-op	*p* value	Mean score change
Lap	6.06	9.24	*<0.001*	3.18 ± 0.69
Robotic	9.77	7.69	*0.023*	−2.14 ± 0.87
*p* value	*0.003*	0.229		*<0.001*

Mean score change from baseline was better in all except one component (initiation/ straining) in the robotic group (see Table 2). Composite mean MUF score change from baseline was also better in the robotic group as illustrated in Table 3 and Fig. 1. Overall, composite mean MUF scores deteriorated in the laparoscopic group and improved in the robotic group (*p* < 0.001, *p* = 0.023), Table 3.

### Male sexual function (MSF)

There were 36 patients (73 %) in the laparoscopic group and 13 (37 %) in the robotic group who were sexually active. Pre-operative scores were similar in both groups. In Tables 4 and 5 and Fig. 1, we present the mean pre-operative MSF scores and their change from baseline.

**Table 4:** Baseline and change from baseline MSF mean scores (mean ± standard error of the mean)

	Laparoscopic	Robotic	*p* value
Baseline MSF			
•Sexually active	Yes 36, no 13	Yes 13, no 22	
•Libido/arousal	0.31	0.54	0.422
•Erection	0.69	0.85	0.712
•Stiffness for penetration	0.86	1.15	0.547
•Orgasm/ejaculation	0.17	0.92	0.057
Change from baseline			
•Libido/arousal	1.56 ± 0.28	0 ± 0.30	*0.001*
•Erection	1.53 ± 0.29	0 ± 0.20	*<0.001*
•Stiffness for penetration	1.39 ± 0.29	−0.38 ± 0.21	*<0.001*
•Orgasm/ejaculation	1.78 ± 0.31	−0.15 ± 0.25	*<0.001*

**Table 5:** Mean composite MSF scores

	Pre-op	Post-op	*p* value	Mean score change
Lap	2.31	8.56	*<0.001*	6.25 ± 1.08
Robotic	3.62	2.92	0.759	−0.69 ± 0.85
*p* value	0.319	*0.004*		*<0.001*

Mean scores deteriorated across all component of MSF in the laparoscopic group but none in the robotic group. Statistical comparison of the mean change of MSF scores from baseline revealed favourable outcomes for the robotic group across all four components of MSF as shown in Table 4. Composite MSF score change was also better in the robotic group as illustrated in Table 5 and Fig. 1.

### Female urological function

There were 29 patients in the laparoscopic group and 13 in the robotic group. The mean pre-operative FUF scores of both groups are shown in Tables 6 and 7. Pre-operative scores were similar in both groups across all components.

**Table 6:** Baseline and change from baseline FUF mean scores (mean ± standard error of the mean)

	Laparoscopic	Robotic	*p* value
Baseline FUF			
•Frequency	1.66	2.23	0.325
•Nocturia	1.79	2.85	0.056
•Urgency	0.76	1.46	0.190
•Stress incontinence	1.10	1.92	0.143
Change from baseline			
•Frequency	0.62 ± 0.25	−0.54 ± 0.56	0.077
•Nocturia	0.69 ± 0.27	0.38 ± 0.40	0.533
•Urgency	0.48 ± 0.16	−0.15 ± 0.27	0.059
•Stress incontinence	0.10 ± 0.11	−0.23 ± 0.28	0.287

**Table 7:** Mean composite FUF scores

	Pre-op	Post-op	*p* value	Mean score change
Lap	5.31	7.21	*<0.001*	1.90 ± 0.50
Robotic	8.46	6.23	0.065	−2.23 ± 1.10
*p* value	0.052	0.501		*0.003*

The mean FUF score changes from baseline are outlined in Tables 6 and 7 and illustrated in Fig. 1. There was no statistical difference in any of the mean FUF component score change from baseline between the two groups. However, composite mean FUF score change from baseline was better in the robotic group. Composite mean FUF score deteriorated in the laparoscopic group but not in the robotic group (see Table 7).

### Female sexual function

There were nine (31 %) sexually active patients in the laparoscopic group and four (31 %) in the robotic group. Their pre-operative scores were similar in both groups as shown in Tables 8 and 9.

**Table 8:** Baseline and change from baseline FSF mean scores (mean ± standard error of the mean)

	Laparoscopic	Robotic	*p* value
Baseline FSF			
•Sexually active	Yes 9, no 20	Yes 4, no 9	
•Arousal/ libido	0.89	1.50	0.589
•Lubrication	1.44	1.25	0.865
•Orgasm	0.44	2.25	0.177
•Dyspareunia	0.44	0.75	0.620
Change from baseline			
•Arousal/ libido	0.67 ± 0.37	−0.25 ± 0.25	0.066
•Lubrication	0 ± 0.17	1.25 ± 0.95	0.279
•Orgasm	0.89 ± 0.42	0 ± 0	0.069
•Dyspareunia	0.44 ± 0.47	2 ± 0.82	0.159

**Table 9:** Mean composite FSF scores

	Pre-op	Post-op	*p* value	Mean score change
Lap	3.22	5.22	0.154	2 ± 1.27
Robotic	5.75	8.75	0.181	3 ± 1.73
*p* value	0.251	0.269		0.657

Mean FSF score changes from baseline are outlined in Tables 8 and 9 and Fig. 1. Overall, there was no statistical difference between the mean change of scores from baseline between the two groups in any of the FSF components or the FSF composite scores.

## Discussion

Robotic rectal surgery has been gaining popularity over the last few years. However, whether it is superior to laparoscopic rectal surgery remains an open debate. In this study, we have found that the robotic approach offers favourable post-operative urogenital functional outcomes in men and urological outcomes in women. Whilst composite MUF, MSF and FUF scores deteriorated in the laparoscopic group, this was not the case for the robotic group. Mean composite score change from baseline for MUF, MSF and FUF favoured the robotic group. The functional score change from baseline was also statistically better in all four components of MSF and in five out of six components in MUF.

Favourable post-operative male sexual function for robotic TME as compared to laparoscopic TME has been demonstrated in previous studies [17, 26–29]. Park et al’s [26] study showed that MSF recovers faster in the robotic group (6 vs 12 months), and at 6 months, the overall MSF scores showed a significantly smaller decrease from baseline in the robotic group (*p* = 0.03). Kim et al. [27], Park et al. [28], D’Annibale et al. [29] and Morelli et al. [17] all demonstrated favourable male sexual outcomes for the robotic group but unlike ours and Park et al’s [26] studies, failed to demonstrate a change of overall MSF scores from baseline in favour of the robotic group. It is worth noting that regarding sexual function, the sample size of these studies was similarly small as to our study, with the range of patients varying between 20 and 14 in the robotic group and 23 and 15 in the laparoscopic group. This shows that a relatively small sample size is sufficient to demonstrate the superiority of robotic rectal surgery in terms of male post-operative sexual function outcomes.

Unlike sexual function, advantages in male urological function following robotic surgery have been harder to demonstrate. A multitude of studies comparing the MUF of robotic vs laparoscopic patients showed no difference in their outcomes [17, 28, 29]. In contrast to the above, Cho et al. [30] in a retrospective study of 278 patients in each group found that at 1 month after surgery, the voiding dysfunction rate was higher in the laparoscopic group (4.3 vs 0.7 %, *p* = 0.012). However, this study did not apply any functional scores for the assessment of dysfunction, leaving it open to observation bias. Park et al. [26] on a population of 32 patients in each group found that at 12 months following surgery, MUF score change from baseline was lower in the robotic group, but this was not quite statistically significant (*p* = 0.051). However, Kim et al. reported a clear advantage for robotic TME in terms of urological function [27]. On a sample size of 30 robotic and 39 laparoscopic patients, they showed that urological function recovered faster in the robotic group (3 vs 6 months) and functional score change from baseline was lower in the robotic group at 3 months (*p* = 0.036). However, we should note that for this study, male and female data were combined. Our study is the first one to demonstrate a significant favourable overall score change from baseline for the robotic approach in a male cohort only. This is probably due to our study’s larger sample size (49 laparoscopic vs 35 robotic).

It is also worth noting that the composite MUF mean score improved in the robotic group. Obviously, we are not suggesting that robotic rectal surgery might itself improve MUF. We believe this might have occurred due to a number of factors. First of all, our study was open to recall bias, since patients reported their urogenital function retrospectively a minimum of 6 months after their surgery. It is possible that patients that did not suffer from any post-operative urological dysfunction might be more prone to better score their post-operative function. Furthermore, despite the scores being based on validated standardised questionnaires, questionnaires themselves are subjective in nature. Ideally, objective measurement tools such as urodynamic studies used in Kim et al’s study [27] should be used in conjunction with questionnaires to increase the validity of the results.

The results from our study indicate favourable outcomes following robotic TME in relation to FUF. Present evidence of FUF following robotic rectal surgery is extremely limited, with only two studies to date investigating FUF independently to that of males [17, 31]. Of those two, only one, whose sample size is considerably smaller, used a control group (laparoscopy) against its robotic cases and found no difference in outcome between the two groups [17].

Regarding FSF, there was no difference between the two groups in any of the individual components or the composite score. However, due to a large proportion of females being sexually inactive, the sample size of the FSF comparison was very small (nine laparoscopic vs four robotic). This makes any meaningful statistical comparison very difficult. Current evidence on robotic FSF is also extremely limited, being investigated in only three studies [17, 31, 32] of which only one used a control group (laparoscopy). In that study, Morelli et al. [17] found no difference in FSF scores between the two groups.

Our study is unique as it has the biggest sample size of its kind. In addition, it is the only study of its kind conducted in the UK, where patient socio-economic background would have no influence on the mode of surgery chosen, since all patients would have been operated in a public healthcare service, the NHS.

In contrast to the majority of the previously published literature, the operating surgeons in our study performed fully robotic rather than hybrid procedures [19]. It is possible that the difference in approach could influence the results. This is because damage to the autonomic nerves could occur in two places where dissection is performed laparoscopically during the hybrid procedure. The superior hypogastric plexus can be damaged during dissection around the inferior mesenteric artery pedicle and the hypogastric nerves during mobilisation of the rectosigmoid colon from the gonadals and the ureter [4, 33]. Therefore, in our study, we exploit the full potential of the robotic approach.

In addition, all the procedures in both the laparoscopic and robotic cohort were performed by the same two surgeons, thus eliminating the confounding factor of the operating surgeon. Moreover, all the laparoscopic procedures pre-dated the robotic ones, since the operating surgeons shifted their regular practice from laparoscopic to robotic TMEs. It could be argued that any skills acquired during the laparoscopic procedures were transferrable to the robotic ones, implying that any superiority demonstrated for the robotic group was not a result of the surgical approach but due to the advancement of the surgeon’s skills. However, this is unlikely considering both surgeons were very experienced laparoscopic rectal surgeons that were trainers for the National Training Programme for Laparoscopic Colorectal Surgery (LAPCO) in the UK.

We acknowledge this study is retrospective and non-randomised in nature. However, despite the lack of randomisation, our results demonstrate from the baseline characteristics that the two groups were broadly comparable. The only differences between the two groups (tumour high, neoadjuvant radiotherapy and chemotherapy) are more likely to negatively skew the results against the robotic group, since long-course radiotherapy and lower rectal tumours possess risks for urogenital dysfunction.

A limitation of our study was that post-operative urogenital function reporting was taken as a “snapshot”, with patients reporting their urogenital outcomes only once in a post-operative period varying from 6 months to 3 years in the laparoscopic group and 8 months to 2 years to in the robotic group. However, unlike other studies, this study does not make any assumptions on the time of recovery of urogenital function but only on the overall post-operative urogenital outcomes.

In summary, our study has demonstrated that robotic rectal cancer surgery might offer favourable overall post-operative urological and sexual outcomes in males and urological outcomes in females. This is probably because robotic systems allow for precise dissection across the surgical planes in narrow spaces such as the pelvis, thus enabling preservation of the pelvic autonomic nerves. We acknowledge that there are limitations in the study’s design such as being retrospective in nature and open to recall bias. We believe a prospective randomised control trial, focusing on urogenital function, with a bigger sample size that includes urodynamic assessment of urological function is required to establish whether robotic surgery truly offers superior post-operative urogenital outcomes.
